# Integrating thermodynamic and sequence contexts improves protein-RNA binding prediction

**DOI:** 10.1371/journal.pcbi.1007283

**Published:** 2019-09-04

**Authors:** Yufeng Su, Yunan Luo, Xiaoming Zhao, Yang Liu, Jian Peng

**Affiliations:** 1 Department of Computer Science, University of Illinois at Urbana-Champaign, Urbana, Illinois, United States of America; 2 School of Electronic Information and Electrical Engineering, Shanghai Jiao Tong University, Shanghai, China; La Jolla Institute for Allergy and Immunology, UNITED STATES

## Abstract

Predicting RNA-binding protein (RBP) specificity is important for understanding gene expression regulation and RNA-mediated enzymatic processes. It is widely believed that RBP binding specificity is determined by both the sequence and structural contexts of RNAs. Existing approaches, including traditional machine learning algorithms and more recently, deep learning models, have been extensively applied to integrate RNA sequence and its predicted or experimental RNA structural probabilities for improving the accuracy of RBP binding prediction. Such models were trained mostly on the large-scale *in vitro* datasets, such as the RNAcompete dataset. However, in RNAcompete, most synthetic RNAs are unstructured, which makes machine learning methods not effectively extract RBP-binding structural preferences. Furthermore, RNA structure may be variable or multi-modal according to both theoretical and experimental evidence. In this work, we propose ThermoNet, a thermodynamic prediction model by integrating a new sequence-embedding convolutional neural network model over a thermodynamic ensemble of RNA secondary structures. First, the sequence-embedding convolutional neural network generalizes the existing k-mer based methods by jointly learning convolutional filters and k-mer embeddings to represent RNA sequence contexts. Second, the thermodynamic average of deep-learning predictions is able to explore structural variability and improves the prediction, especially for the structured RNAs. Extensive experiments demonstrate that our method significantly outperforms existing approaches, including RCK, DeepBind and several other recent state-of-the-art methods for predictions on both *in vitro* and *in vivo* data. The implementation of ThermoNet is available at https://github.com/suyufeng/ThermoNet.

## Author summary

RNA-binding proteins (RBPs) play a key role in modulating various cellular processes, including transcription, alternative splicing, and translational regulation. Identifying protein-RNA interactions and the binding preferences of RBPs are critical to unraveling the mechanism of post-transcriptional gene regulation. In the current study, we present a computational approach that integrates both structure and sequence contexts for protein-RNA binding prediction. We propose to incorporate the structure information using a thermodynamic ensemble of secondary structures, which effectively identifies RBP-binding structural preferences, especially for structured RNAs. Our model is further empowered by a deep neural network that combines the sequence and structure information to achieve improved protein-RNA binding prediction. Extensive experiments on both *in vitro* and *in vivo* datasets demonstrate the superior performance of our method compared to several state-of-the-art approaches. This study suggests the great potential of our method as a practical tool for identifying novel protein-RNA interactions and binding sites of RBPs.

## Introduction

RNA-binding proteins (RBPs) modulate the processing of cellular RNAs, including their production, transportation, splicing, stability, translation, and degradation [1, 2]. There are more than 1,500 RBPs in the human genome which are identified with well-defined RNA-binding domains (RBDs) [3], including the RNA recognition motif (RRM) [4], the K-homology domain (KH) [4], and the C3H1 zinc-finger (ZF) domain [5]. Existing studies on different RNA binding domains indicate that their interaction specificities with RNAs are determined by various sequence- and/or structure-specific patterns. For example, sequence motifs on hairpins or loops are more accessible to many RBPs, while some RBPs, such as RBM22, RBM6 and PRR3, show a structural preference on bulged stems [6]. Understanding of the sequence/structure specificity of RNA-binding proteins is therefore critical for developing hypotheses and models of post-transcriptional gene regulation [7].

*In vitro* and *in vivo* methods have been developed for determining RBP binding specificities. RNAcompete is a high-throughput *in vitro* assay that quantifies the relative affinity of a specific RBP to a pre-defined set of more than 240,000 short RNAs. In a recent work, more than 200 human RBPs are analyzed by RNAcompete, generating the first large-scale dataset of protein-RNA interaction measurements [8]. A major limitation of RNAcompete is that the designed RNAs are only 41nt long and not structurally stable, therefore the motifs identified by this approach are predominantly in short unstructured contexts. SELEX (systematic evolution of ligands by exponential selection) [9] iteratively selects a set of high-affinity RNA sequences from a large pool, similar to the evolutionary procedure used in optimization, which is generally biased or suffers from undersampling. Different from *in vitro* methods, high-throughput *in vivo* techniques have been developed to measure genome-wide RBP-RNA interactions in their cellular contexts. For example, CLIP-seq, RIP-seq and their variants [10, 11] provide high-resolution protein-RNA binding sites. However, it is usually not easy to clearly derive binding motifs from these experiments, because of the existence of protein cofactors, technical artifacts, RNA folding, and high levels of noise or non-specific background. Therefore, learning algorithms for computational prediction of protein-RNA binding from *in vitro* data may be less affected by the noise and provide insights that can be generalized to *in vivo* data.

Different computational methods have been introduced to protein-RNA binding prediction. Traditionally, the sequence specificities of an RBP is most commonly modeled by position weight matrix (PWM) [12] or hidden Markov models, which are solely based on the biases or enrichments of nucleotides on the binding sites. Such models can be learned from a collection of RNA sequences with high binding affinities. To take RNA structure into consideration, MEMERIS [13] applies MEME [14, 15] to identify binding sites in unpaired loop regions. RNAcontext [16] learns a joint probabilistic model of both sequence and structure contexts. RCK [17], a recent improvement of RNAcontext, uses *k*-mer based contexts incorporating both sequence- and structure-based binding preferences. More recently, deep learning methods have been adapted to protein-RNA binding prediction. DeepBind [18] and DLPRB [19] utilize convolutional neural networks (CNN) to jointly extract binding preferences from both RNA sequence and structure and demonstrate substantial improvements, compared to previous approaches.

In both deep learning approaches, DeepBind [18] and DLPRB [20], RNA sequence contexts are modeled by a small number of convolutional filters, each resembling a *k*-mer or a binding site. RNA structural contexts are represented as a probability matrix for structure types (paired (P), hairpin loop (H), inner loop (I), multi-loop (M) or external region (E)) [16], each measuring the thermodynamic average of a structure type of the full ensemble of all possible structures. This matrix can be computed by an RNA folding algorithm with dynamic programming [21]. Models in both DeepBind and DLPRB were trained on the large-scale *in vitro* RNAcompete dataset. However, in RNAcompete, most synthetic RNAs are unstructured, which makes deep learning methods not effectively identifying RBP-binding structural preferences. Furthermore, RNA structure may be variable or multi-modal according to both theoretical and experimental evidence. In this work, we propose ThermoNet, a thermodynamic prediction model by integrating a new sequence-embedding convolutional neural network model over a thermodynamic ensemble of RNA secondary structures. First, our sequence-embedding convolutional neural network generalizes the existing *k*-mer based methods by jointly learning convolutional filters and *k*-mer embeddings to represent RNA sequence contexts. In this way, each *k*-mer is represented by a low-dimensional continuous vector, and convolutional filters combine a set of *k*-mer embeddings of consecutive positions and their corresponding structural contexts, thus providing more flexibility and higher expressiveness. Second, the thermodynamic average of structure-specific predictions explores structural variability and improves the prediction especially for the structured RNAs in *in vivo* datasets. Structural contexts in the high-probability structures are not simply averaged but used for providing structure-specific predictions. Extensive experiments demonstrate that our method significantly outperforms existing approaches, including RCK, DeepBind, DLPRB and several other recent state-of-the-art methods for predictions on both *in vitro* and *in vivo* data.

## Materials and methods

**Fig 1:**
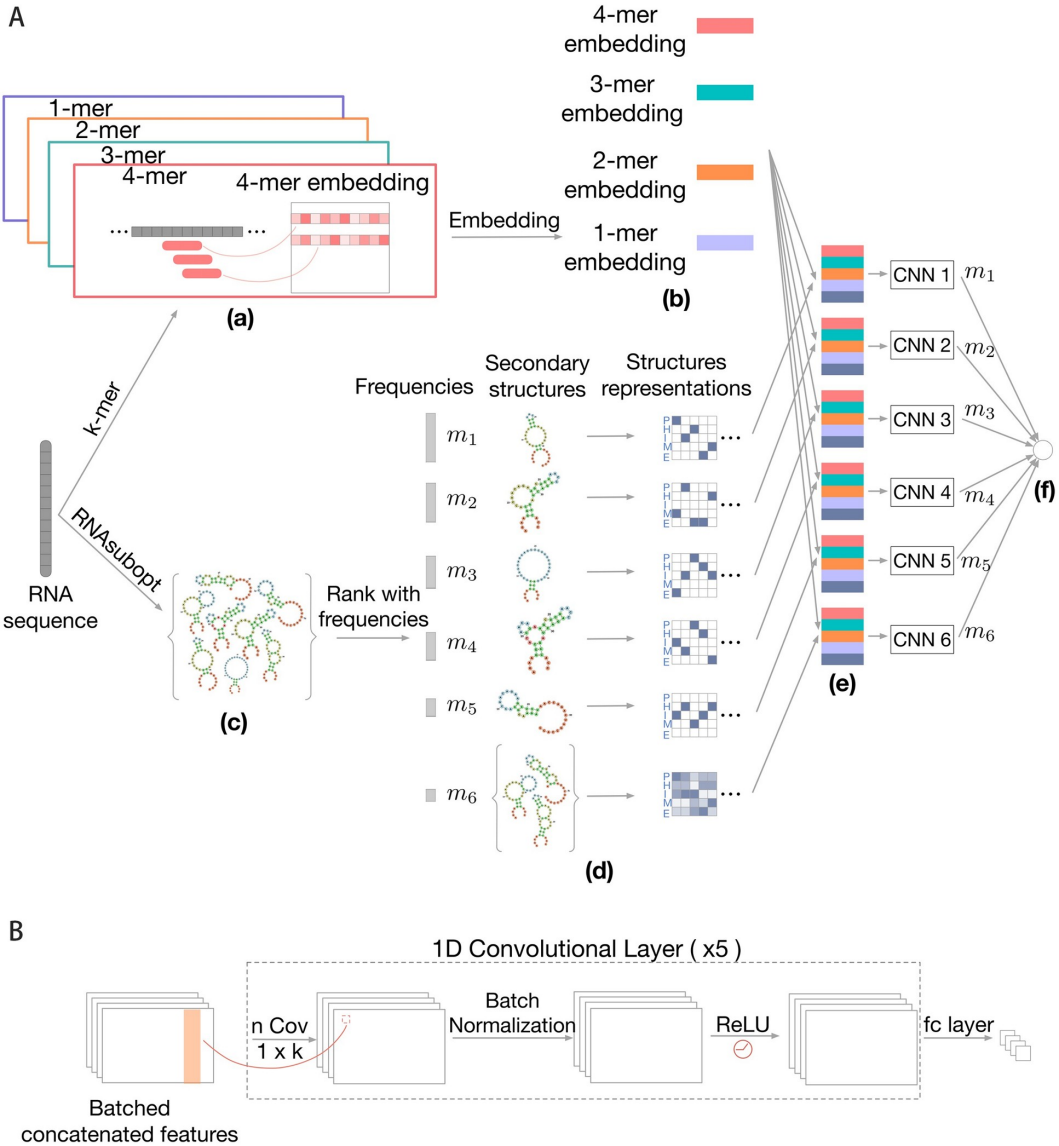
(A) **Overall framework of ThermoNet**. (a) Our model receives the RNA sequence as the input and *k*-mers with various lengths are extracted as sequence features. (b) Each *k*-mer is mapped to a low-dimensional continuous vector (called sequence embedding) through an embedding layer to construct a sequence representation of the input sequence. (c) An ensemble of possible secondary structures of the input RNA are sampled using the RNAsubopt function of Vienna RNA package. (d) High-probability structures from the structure ensemble are encoded using one-hot representation separately and the remaining structures are summarized by averaging their one-hot representations into an average structure profile. (e) Each of the high-probability structures is integrated with the sequence embedding by a convolutional neural network (CNN) to produce structure-specific predictions. The average structure profile is also combined with the sequence embedding to generate a prediction. (f) The final predicted protein-RNA binding intensity is obtained by computing the weighted average of individual predictions. (B) **Prediction network structure**. The convolutional neural network takes the sequence embedding and secondary structure representation as input. The network is composed of multiple 1D convolutional kernels, followed by a batch normalization layer, a ReLU activation layer and fully-connected (fc) layer. The output of the network is the predicted binding intensity for the input RNA.

We introduce a deep learning-based thermodynamic prediction model for protein-RNA binding prediction. The model integrates both sequence information and structural contexts to better capture RBP-binding preferences. As an overview (Fig 1), our method takes an RNA sequence as input and extracts informative features from the sequence and a thermodynamic ensemble of its corresponding secondary structures. A deep convolutional neural network is used to integrate the sequence and structure information and produce the prediction of binding affinity. We have two main contributions here: first, we use a sequence-embedding convolutional neural network (CNN) to represent each *k*-mer as a low-dimensional continuous vector, which is more flexible and expressive than previous methods that directly apply CNN on nucleotides [18, 22] or solely use traditional *k*-mer based features [17]; second, we use a thermodynamic ensemble of RNA secondary structures to explore the structural variability and provide structure-specific predictions—this differentiates our method from previous approaches that simply collapse the set of (predicted) secondary structures by taking the average, in which the structure-specific information of high-probability structures may be mixed. We describe the details of our method below.

### Input sequence and structure representations

An RNA sequence of length *ℓ* is a string of *ℓ* nucleotides over the alphabet {A, C, G, U}. We represent the sequence using the one-hot encoding scheme, where A, C, G and U are represented by [1, 0, 0, 0], [0, 1, 0, 0], [0, 0, 1, 0], and [0, 0, 0, 1], respectively. The secondary structure of the RNA is a string of the same length *ℓ* over the alphabet {P, H, I, M, E}, representing the five types of structural contexts, namely, paired (P), hairpin loop (H), inner-loop (I), multi-loop (M) or external region (E). Existing approaches, including DeepBind [16, 18], DLPRB [20] and RCK [17], represent the structural context of a position as a five-dimensional distribution vector corresponding to the probabilities of each type of structural contexts predicted by an RNA folding algorithm such as RNAplfold [21]. If only a specific structure is considered, the five-dimensional vector becomes one-hot.

### Extracting sequence context features using *k*-mer embeddings

To better extract informative features from the RNA sequence, we propose a sequence-embedding convolutional neural network that first augments sequence features with *k*-mers and then uses a convolutional neural network (CNN) to extract higher-order features.

It has been demonstrated that incorporating *k*-mer based features, in addition to nucleotide based features, can encode larger sequence contexts, model dependencies between binding site positions and thus improve the prediction performance [17]. Here we generalize this approach to account for *k*-mers with various lengths (e.g., *k* = 1, …, 5). The straightforward way is to simply concatenate *k*-mers with various lengths together and encode it using one-hot representation. However, the dimensionality of this representation increases exponentially (4^*k*^), which poses computational challenges in the prediction. To address this, we apply the embedding layer that is widely used in deep learning to do dimensionality reduction. The embedding layer can be thought of as a look-up table, which maps the 4^*k*^-dimensional one-hot vector to a *d*-dimensional continuous vector. The values of the *d*-dimensional vector are not pre-specified but rather learned from the data during the model training process. For an input RNA sequence of length *ℓ* and a fixed *k*-mer length *k*, the output of the embedding layer is a matrix with dimension *ℓ* × *d*, in which the *i*-th row is the low-dimensional representation of the *k*-mer starting at the position *i* (*k*-mers go beyond the RNA sequence length are zero-padded). To account for various *k*-mer lengths (i.e., *k* = 1, …, *k*_*m*_), we stack the representations of each *k*-mer length to produce a sequence representation matrix **E** with dimension *ℓ* × *k*_*m*_*d* where *k*_*m*_*d* ≪ 4^*k*^. These low-dimensional representations of *k*-mers will be integrated by a CNN to further capture the higher-order dependencies in the RNA sequence (described below).

Compared with existing approaches, our method has more flexibility and higher expressiveness in extracting sequence features: Unlike RCK [17] that uses *k*-mer based features with only a specific length (*k* = 5), our method is more flexible in that it can handle various lengths of *k*-mers through a dimensionality reduction process, capturing multi-resolution local sequence information. cDeepbind [22] directly applies CNN on nucleotide based input features, while our method incorporates *k*-mer based features and then uses a CNN on top of the *k*-mer embeddings to further extract higher-order features, providing richer information from the RNA sequence.

### Incorporating thermodynamics contexts using an ensemble of structures

Previous studies have shown that RNA secondary structure information can provide additional prediction power in predicting protein-RNA binding [17, 20, 22]. These methods use the RNA secondary structure context profiles predicted by RNAplfold [16, 21], which is a five-dimensional vector of the probabilities of five contexts (paired, hairpin loop, inner loop, multi-loop or external region) for each position in the sequence. The structure context profile can be thought of as an ‘average’ of all possible secondary structures. However, informative structural features of high-probability structures may be buried in this uniform average. To enable structure-specific prediction, we propose to sample an ensemble of possible structures for a given RNA sequence. Each of the high-probability structures is then integrated with the sequence features to give structure-specific predictions separately, and the final prediction is obtained by combining the structure-specific predictions.

Specifically, for a given RNA sequence, we sample *N* possible secondary structures using the RNAsubopt function of the ViennaRNA package [21], which draws a specific structure with probability proportional to its Boltzmann energy. In our experiment, we choose *N* = 100 for computational consideration and we also found that an ensemble of this size generally reflects the distribution of the secondary structures of an RNA sequence. We then identify high-probability structures by counting the occurrence for each unique structures of the *N* sampled structures. Denote the *U* unique structures by **R**_1_, **R**_2_, …, **R**_*U*_ and their associated frequency by *m*_1_, *m*_2_, …, *m*_*U*_, where **R**_*i*_ (1 ≤ *i* ≤ *U*) is a *ℓ* × 5 one-hot matrix that represents the structural contexts of the *i*-th sampled structure. Here we assume the unique structures are sorted in descending order of their frequencies, i.e., *m*_1_ ≥ *m*_2_ ≥ …, ≥*m*_*U*_. The top *T* structures are considered as high-probability structures. The choice of the value of *T* is a joint consideration of structures diversity and computational cost: we want to include more high-probability structures, while also prevent *T* being too large otherwise the model training and prediction would be inefficient. Based on the analysis of the frequency histogram, we found *T* = 5 is a good balance between structures diversity and computation efficiency for both *in vitro* and *in vivo* datasets (S1 Fig and S1 Table). For example, in the *in vitro* dataset, the top 5 unique structures are highly frequent, contributing ∼50% frequencies in all sampled structures. To reduce noise, we thus consider the top *T* structures as high-probability structures, while for the remaining *U* − *T* structures, we summarize their structure information by computing an average structure profile

$$R_{T+1}=\sum_{i=T+1}^{U}\frac{m_{i}}{\sum_{j=T+1}^{j=U}m_{j}}R_{i}$$ (1)

Next, we build a series of rank-specific neural networks (described below) $f_{\theta_{1}}$, $f_{\theta_{2}}$, …, $f_{\theta_{T}}$, parameterized by *θ*_1_, …, *θ*_*T*_, respectively. Each neural network takes as input one of the high-probability structures **R**_*i*_ as well as the sequence embedding **E** to produce a structure-specific prediction (a binding intensity). We also build an additional neural network $f_{\theta_{T+1}}$ that makes the prediction based on the average structure profile and the sequence embedding. Predictions of each individual neural network are combined as a weighted average to give the final prediction *f*(*s*) for an RNA sequence *s*,

$$f\left(s\right)=\sum_{i=1}^{T}\frac{m_{i}}{N}f_{\theta_{i}}\left(E,R_{i}\right)+(1-\sum_{i=1}^{T}\frac{m_{i}}{N})f_{\theta_{T+1}}\left(E,R_{T+1}\right)$$ (2)

Note that previous methods like cDeepbind [22] make predictions using only the average structure profile as input, in which the structure-specific information may not be revealed. In contrast, our method, while accounting for the average structure profile, also explicitly teases high-probability structures apart from the uniform average profile to enable structure-specific predictions.

### Convolutional neural network as base predictor

Deep learning continues to proliferate as a powerful set of tools to solve an increasingly diverse range of problems, including many in structural and systems biology [23–29]. We use the convolutional neural network (CNN) as the base model *f*_*θ*_ to predict protein-RNA binding, where *θ* represents the network weights. CNNs are useful when local groups of data with high correlation and forming distinct patterns by combining lower level features and capturing more complex feature dependencies in the sequence input. In our model, we stack five convolutional layers. Every layer contains several one-dimensional convolutional kernels, each of which produces an output with the same size of the input. After applying the batch normalization technique [30] to the intermediate output from kernels, we fed it to the ReLU activation function [31] and give the output to the next convolutional layer. Following the last convolutional layer, we append a fully-connected layer to produce the predicted binding intensity of the protein-RNA binding. The overall structure is depicted in Fig 1B. Please refer to Results section for parameter-tuning details. In order to improve the training stability of ThermoNet, we train the *T* + 1 models individually. Each model $f_{\theta_{i}}$ was trained using stochastic gradient descent to find the parameters of the embedding layer and CNNs that minimize the following loss function

$$L_{i}=L_{\text{Huber}}\left(y_{s},f_{\theta_{i}}\left(E,R_{i}\right)\right)$$ (3)

where *y*_*s*_ is the (normalized) binding affinity of the RNA *s* to a given protein, $f_{\theta_{i}}\left(E,R_{i}\right)$ is the predicted binding intensity by the model, and *L*_Huber_() is the Huber loss, which is defined by

$$L_{\text{Huber}}\left(y_{s},f\left(s\right)\right)=\{\begin{array}{cc} \frac{1}{2}{\left(y_{s}-f\left(s\right)\right)}^{2} & \text{if}\;|y_{s}-f\left(s\right)|\leq 1\\ |y_{s}-f\left(s\right)|-\frac{1}{2} & \text{otherwise} \end{array}$$ (4)

We choose Huber loss as it is less sensitive to outlier data points than the squared error loss.

## Results

### Implementation

We performed the grid search to find the optimal hyperparameters of our model and chose the one with the lowest validation cost using a two-fold cross-validation on the training data. In particular, we grid-searched the initial learning rate in {0.001, 0.0001, 0.00001}, the filter lengths in {7, 12, 16}, the number of convolutional kernels in {16, 64}, the regularization coefficient of the *L*_2_ norm in {0.001, 0.0001, 0.00001}, the size of sequence embedding vector in {10, 20, 30} and the maximum *k*-mer length *k*_m_ in {2, 3, 4, 5}. A total of five convolutional layers were stacked in the CNN. All CNN parameters were initialized by Xavier initializer [32]. We chose Huber loss to define the loss function and Adam [33] as the optimizer for training the network. We reduced the learning rate to one-tenth of the previous one after the 5th epoch.

### Evaluation of prediction performance on *in vitro* data

We first assessed the prediction ability of our method using *in vitro* protein-RNA binding dataset. We used the comprehensive RNAcompete dataset [8] which includes binding intensities for over 240,000 sequences across 244 experiments. The dataset consists of a training set of sequences (set A) and a test set (set B). For each experiment, we trained a model on sequences extracted from set A and then predicted the intensities for sequences from set B. To evaluate the performance, we used the Pearson correlation between predicted and measured intensities on set B as the metric. Similar to Deepbind [18], we removed outlier intensities in the dataset: we clamped all intensities greater than the 99.5 percentile to the 99.5 percentile. Furthermore, the scores were normalized to have a mean of zero and a variance of one.

**Fig 2:**
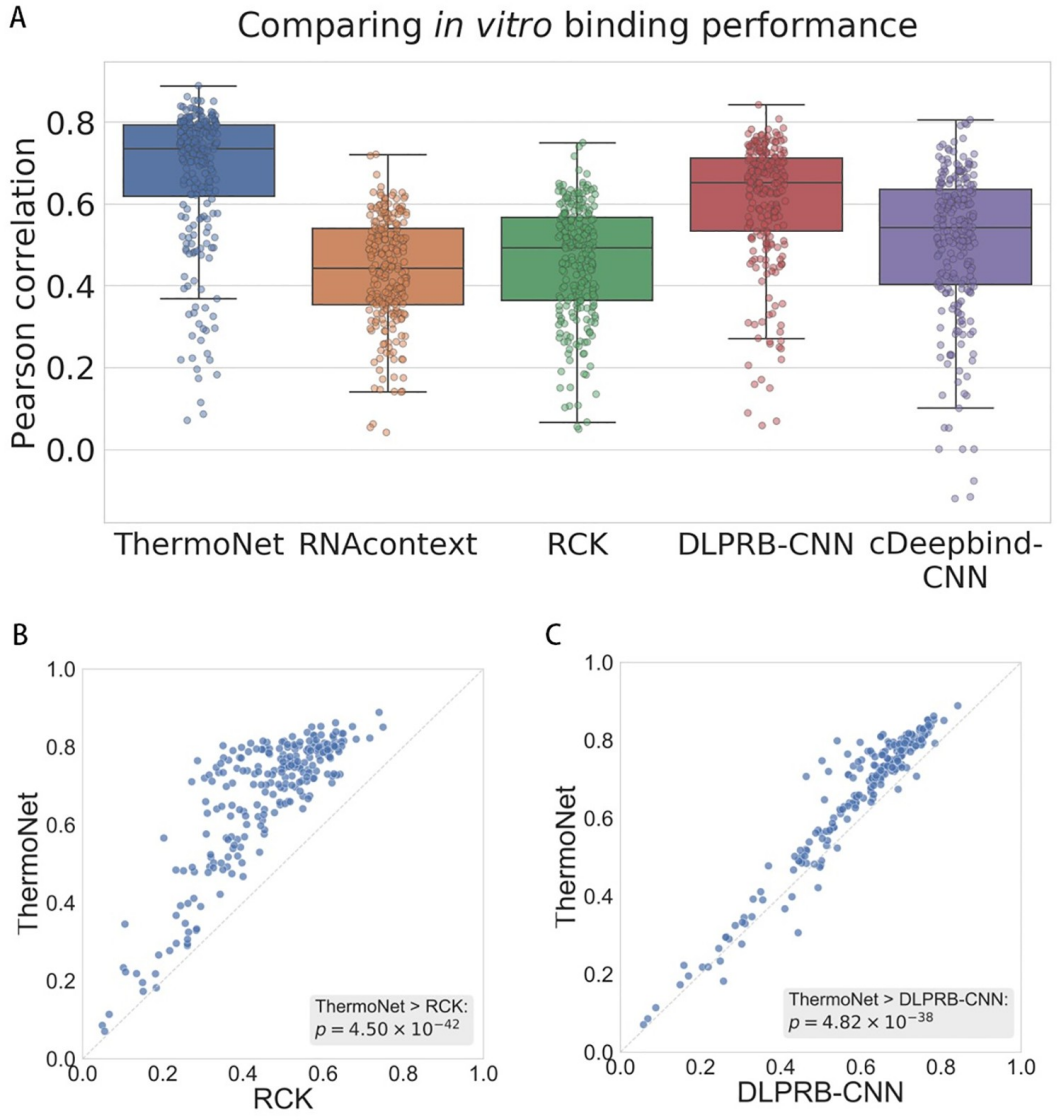
(A) Comparison between ThermoNet and four state-of-the-art methods in predicting *in vitro* binding. Performances were evaluated using the Pearson correlation between the predicted and measured binding intensities. Points in each box plot represent the 244 experiments in the RNAcompete [8] dataset. (B)-(C) Pairwise comparisons of ThermoNet to RCK [17] and DLPRB-CNN [20], respectively. The *p*-values were calculated using one-sided Wilcoxon singed rank test.

We compared our method against four existing methods that use both sequence and structure information for protein-RNA binding prediction, including two probabilistic model based method—RNAcontext [16] and RCK [17], and two deep learning algorithms—DLPRB with the CNN network (DLPRB-CNN) [19] and cDeepbind with the CNN network (cDeepbind-CNN) [22]. We found that our method consistently outperformed all other methods (Fig 2A) with an average Pearson correlation of 0.6710 over all proteins, compared to 0.4344, 0.4600, 0.6058 and 0.5061 for RNAcontext, RCK, DLPRB-CNN and cDeepbind-CNN, respectively. The relative improvements achieved by our method over the for methods were 54.46%, 45.87%, 10.76% and 32.58%, respectively. In a pairwise comparison to the best performing probabilistic model RCK and the best performing deep learning model DLPRB-CNN (Fig 2B and 2C), we observed that our method achieves significantly higher Pearson correlation than the two baseline methods (Wilcoxon signed rank test *p*-value 4.50 × 10^−42^ and 4.82 × 10^−38^, respectively). Moreover, we found that the improvements achieved by ThermoNet are not biased toward certain predominant RNA binding domains (S2 and S3 Figs). For example, investigating the histogram of relative improvements achieved by our method (S4 Fig), we found that the subset of RBPs with the most noticeable improvements covered a wide range of RNA binding domains. Raw numbers of prediction performance of all methods in this experiment can be found in S2 Table.

### Evaluation of prediction performance on *in vivo* data

We then assessed the performance of ThermoNet on *in vivo* protein-RNA binding dataset. We obtained a large-scale dataset of RBP binding sites from a previous work [34], including a compendium of 31 published CLIP-seq experiments on 19 RBPs. In this dataset, nucleotide positions with the highest cDNA counts were first identified as a pool of positive samples. A random sampling process preventing consecutive genomic positions was then applied to reduce the redundancy. Negative sites were randomly sampled from genes that were not detected as positive sites in any of the 31 experiments. Each experiment consists of 40,000 samples divided into a training set of 20,000 samples and a test set of 20,000 samples. The length of each RNA sequence is 101nt. Unlike the *in vitro* dataset that gives a real-valued binding intensity for every binding event, the *in vivo* dataset only gives a binary label (0 or 1) based on the cDNA counts to indicate whether a protein binds to an RNA or not. Therefore, we treated the prediction for *in vivo* data as a binary classification problem and used AUROC (Area Under the Receiver Operating Characteristics) as the evaluation metric.

**Fig 3:**
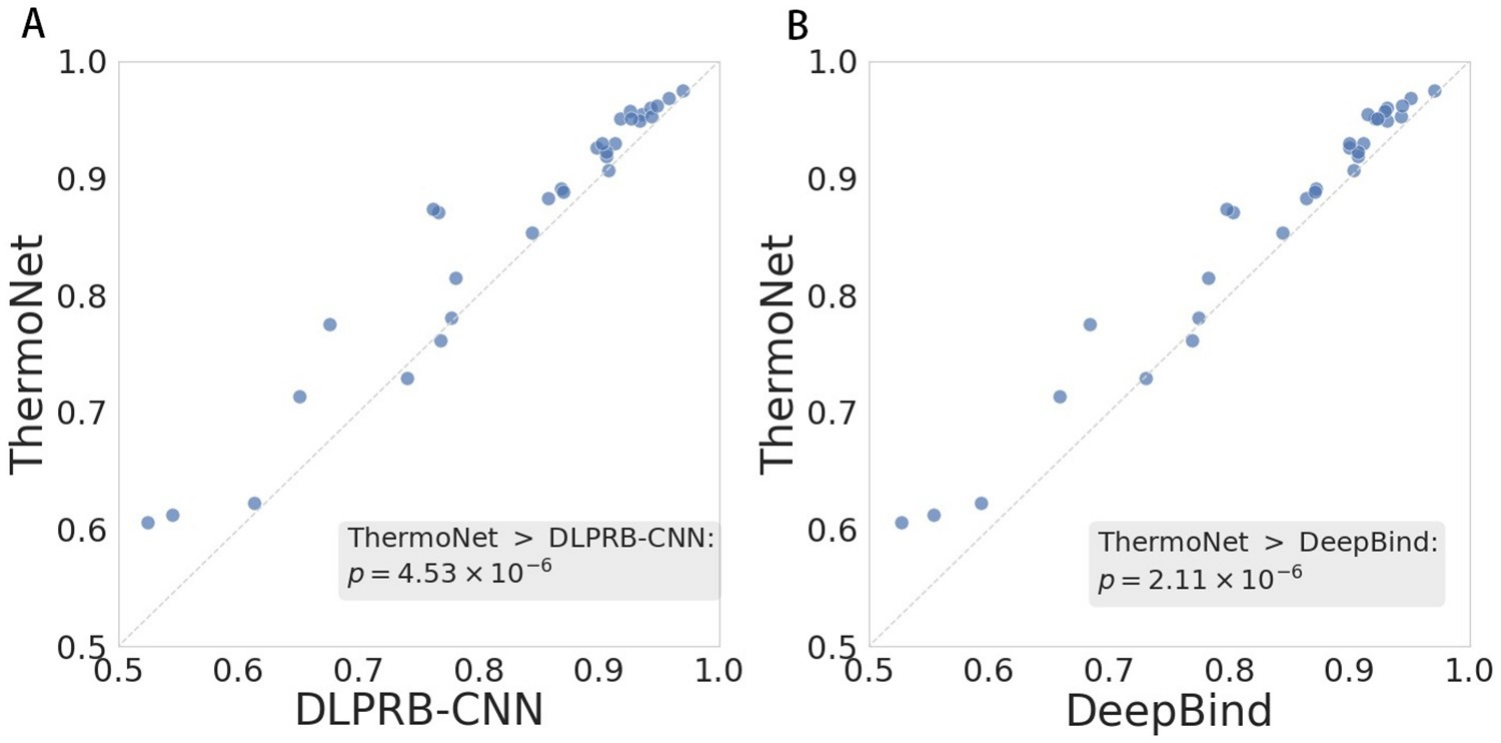
Pairwise comparisons of ThermoNet to (A) DLPRB-CNN and (B) DeepBind in *in vivo* binding. AUROC was used as the evaluation metric. Each point in the scatter plot represents one of 31 CLIP-seq experiments. The *p*-values were calculated using one-sided Wilcoxon singed rank test.

We compared ThermoNet against two state-of-the-art deep learning algorithms, DeepBind and DLPRB-CNN (Fig 3), using the average of the AUROCs over the 31 experiments as the evaluation metric. We found that ThermoNet outperformed the other two methods, achieving an average AUROC of 0.864 against 0.835 for DLPRB-CNN and 0.836 for DeepBind. In a pairwise comparison of ThermoNet to DeepBind and DLPRB-CNN over the 31 experiments, we observed that the improvement gaps between ThermoNet and the two methods were also statistically significant (Wilcoxon signed rank test *p*-value 4.53 × 10^−6^ and 2.11 × 10^−6^ for DLPRB-CNN and DeepBind, respectively). Raw numbers of prediction performance of all methods in this experiment can be found in S3 Table.

### Ablation analysis of ThermoNet

**Fig 4:**
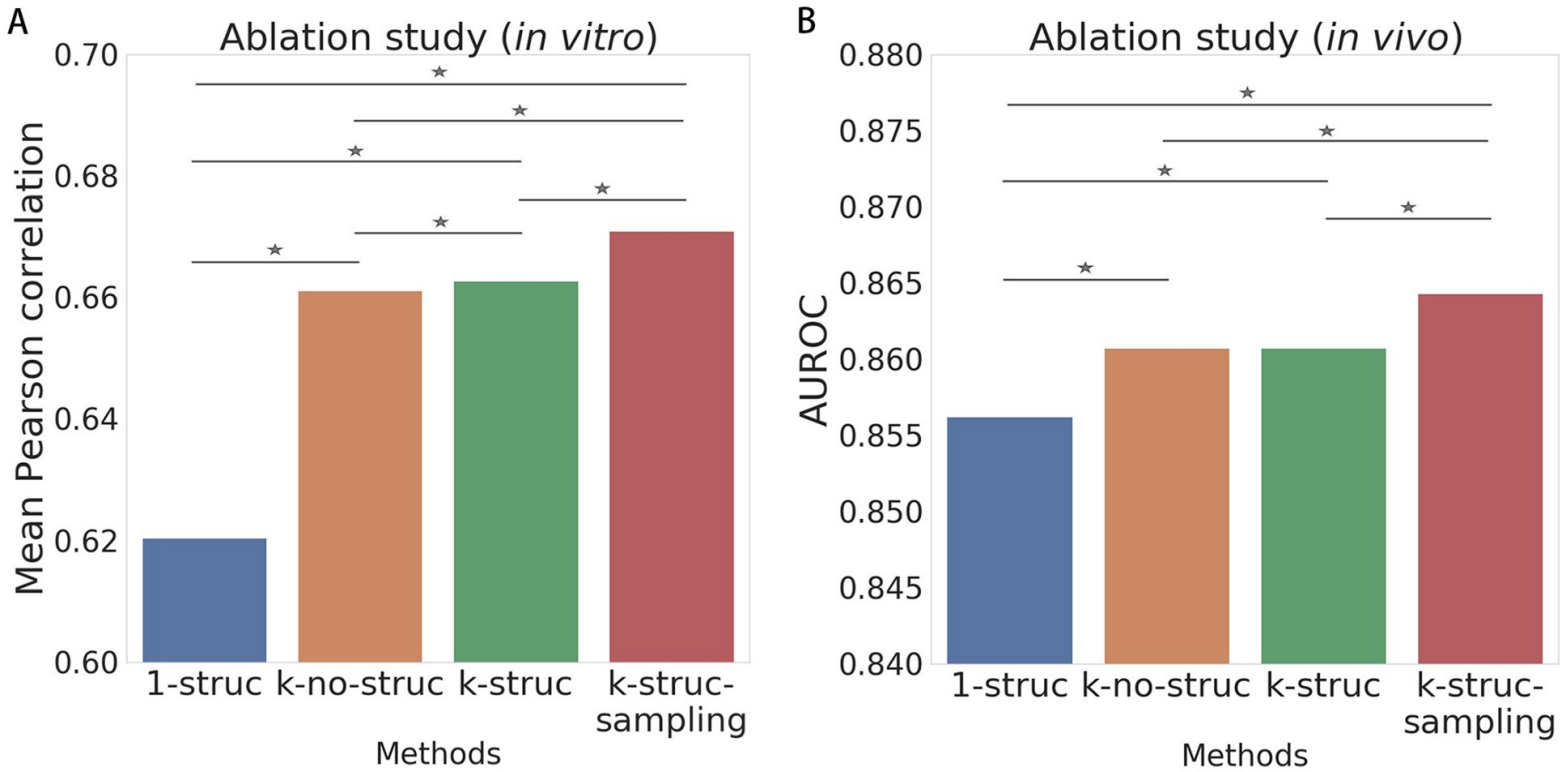
Ablation analysis of various designs in ThermoNet using (A) *in vitro* and (B) *in vivo* datasets. Several variants of ThermoNet were built and evaluated, including *1-struc* (blue): training only one CNN with 1-mer representations and averaged secondary structure information. *k-no-struc* (brown): training only one network with various *k*-mer representations but without any structure information. *k-struc* (green): training only one CNN with various *k*-mer representations as well as averaged structure information. *k-struc-sampling* (red): training various networks for individual secondary structures. All variants received the sequence embedding settings as input. ⋆: one-sided Wilcoxon signed rank test *p*-value < 0.05.

Having demonstrated the superior prediction ability of ThermoNet for both the *in vitro* and *in vivo* bindings, we proceeded to perform an ablation analysis to explain the sources of performance improvements achieved by ThermoNet. We built several variants of ThermoNet to investigate the importance of different novel designs in ThermoNet. The variants include i) “*1-struc*”: using only 1-mer and the average structure profile as input; ii) “*k-no-struc*”: using *k*-mers of various lengths and without using structure information as input; iii) “*k-struct*”: using *k*-mers of various lengths and the average structure profile as input; and iv) “*k-struc-sampling*”: the full model, i.e., using *k*-mers of various lengths, individual secondary structures and average structure profile as input. All variants were trained and tested on the aforementioned *in vitro* dataset. We evaluated the performance of each variant using the average Pearson correlation over the 244 experiments in the *in vitro* dataset and the 31 experiments in the *in vivo* dataset (Fig 4; Detailed results in S4 Table).

We first observed that the *k-struc* model (green) improves the *1-struc* model (blue) with a pronounced gap (average correlation 0.663 compared to 0.620 for the *in vitro* dataset and AUROC 0.861 compared to 0.856 for the *in vivo* dataset). Note that previous methods such as DeepBind, cDeepbind and DLPRB only used single nucleotide based sequences. The improvements of our method over these methods can thus be partially explained by the richer sequence information we have captured in the *k*-mer embedding process of our method.

In addition, the performance gap between the *k-struc* model (green) and the *k-no-struc* model (brown) was not significant: on the *in vitro* dataset, the *k-struc* model achieved an average correlation of 0.663 as compared to 0.661 for the *k-no-struc* model; on the *in vivo* dataset, both models achieved an AUROC of 0.861. The only difference between these two models is that *k-struc* used the average structure profile but *k-no-struc* did not. As the average structure profile is a uniform average of all sampled secondary structures, informative features of high-probability structures that are useful for protein-RNA binding prediction may not be revealed by the average profile, and thus the performance improvement was not significant. The small performance gap between the two variants may also be explained by the high expressiveness of the sequence embeddings, which already capture the information that the average structure profile can provide for the binding prediction.

Moreover, we found that adding individual high-probability structures as input (the *k-struc-sampling* model) further improved the prediction performance of the *k-struc* model (average correlation 0.671 compared to 0.663 for the *in vitro* dataset and AUROC 0.864 compared to 0.861 for the *in vivo* dataset). This result highlights the effectiveness of using the thermodynamic ensemble of secondary structures in ThermoNet. The unique features of structured RNAs revealed by the high-probability structures and the thermodynamic average of structure-specific predictions have resulted in a substantial improvement over existing methods.

## Discussion

We have introduced ThermoNet, a deep learning-based thermodynamic model for protein-RNA binding prediction. ThermoNet incorporates the thermodynamic and sequence contexts by integrating a sequence-embedding convolutional neural network over a thermodynamic ensemble of RNA secondary structures. The model both explores structural variability and captures the higher-order dependencies in the RNA sequences, providing richer information and higher expressiveness for protein-RNA binding prediction. In addition, high-probability structures are utilized in ThermoNet to better extract informative structure features that enable structure-specific predictions. We compared ThermoNet to multiple state-of-the-art methods for protein-RNA binding prediction on both *in vitro* and *in vivo* binding datasets, and ThermoNet has achieved substantial improvements over other methods on both datasets. Ablation study performed on a series of ThermoNet also demonstrated the effectiveness of multiple novel designs in ThermoNet that lead to improved prediction performance.

ThermoNet is a flexible and scalable model that can be applied to a broad range of RNA-binding proteins. We expect ThermoNet to be an effective tool in practice for identifying novel binding sites for RBPs. In addition to its direct application in protein-RNA binding prediction, multiple novel model designs of ThermoNet can also be used as a stand-alone tool in other applications. For example, the sequence-embedding convolutional neural network can also be applied to understand signals in biological sequence data of other molecular events, including transcription factor (TF) binding, DNA accessibility, and histone modification. Interpreting the ThermoNet model is one of the directions worth pursuing in future work. A better understanding of what the deep learning model has learned, for example, what sequence and structure motifs contribute to protein-RNA binding, may reveal new biological insights. A great challenge of protein-RNA binding prediction is the *in vitro*-to-*in vivo* generalization. It was observed in previous works [17, 20] that a model trained on *in vitro* data did not perform very well on *in vivo* data, possibly due to the biases of different experimental protocols [35]. We think the generalization requires not only a generalizable prediction model but also an in-depth characterization of the *in vitro* and *in vivo* datasets. Achieving a robust *in vitro*-to-*in vivo* generalization in protein-RNA binding prediction is also one of our future directions.

## Supporting information

S1 Table Secondary structures frequency statistics in *in vitro* dataset and *in vivo* dataset. This table is also available at https://github.com/suyufeng/ThermoNet/tree/master/supplementary.
(XLSX)

S2 Table Comparison of ThermoNet against RNAcontext, RCK, DLPRB-CNN and cDeepBind-CNN on *in vitro* binding, measured using pearson correlation. This table is also available at https://github.com/suyufeng/ThermoNet/tree/master/supplementary.
(XLSX)

S3 Table Comparison of ThermoNet against DLPRB-CNN and DeepBind on *in vivo* binding, measured using AUROC. This table is also available at https://github.com/suyufeng/ThermoNet/tree/master/supplementary.
(XLSX)

S4 Table Ablation study for various features on *in vivo* and *in vitro* binding. This table is also available at https://github.com/suyufeng/ThermoNet/tree/master/supplementary.
(XLSX)

S1 Fig Frequency histogram of unique sampled secondary structures in (A) *in vitro* and (B) *in vivo* datasets. Structures are sorted from the most frequent to the least frequent in the x-axis. The y-axis shows the average frequencies of structures across all RNAs in each dataset. Structures ranked at the top *T* = 5 are colored in purple and the remaining structures are colored in yellow.
(TIF)

S2 Fig The distribution of the RNA-binding domains on the *in vitro* dataset (RNAcompete). (TIF)

S3 Fig Pairwise comparisons of ThermoNet to (A) RCK and (B) DLPRB-CNN on the RNAcompete dataset. Each point in the scatter plots represents an experiment in the RNAcompete dataset and is labeled with a color specific to its corresponding RNA-binding domain.
(TIF)

S4 Fig The histogram of relative improvements achieved by ThermoNet. We compute the relative improvement over DLPRB-CNN achieved by our method for each experiment in the RNAcompete dataset. These relative improvements are sorted from the largest to the smallest and then discretized into bins of percentiles (0^*th*^, 10^*th*^], (10^*th*^, 25^*th*^], (25^*th*^, 50^*th*^], (50^*th*^, 100^*th*^]. The normalized counts of each RNA-binding domain within each bin are shown in the histogram. Note that the S1 binding domain has only one protein hence the relative count of the S1 domain in the (0^*th*^, 10^*th*^] bin is 1.0.
(TIF)
