# Supplementary figures and images for: Integrating thermodynamic and sequence contexts improves protein-RNA binding prediction
Source: PLoS Comput Biol. 2019 Sep 4;15(9):e1007283. doi: 10.1371/journal.pcbi.1007283 (PMC6752863; doi:10.1371/journal.pcbi.1007283)

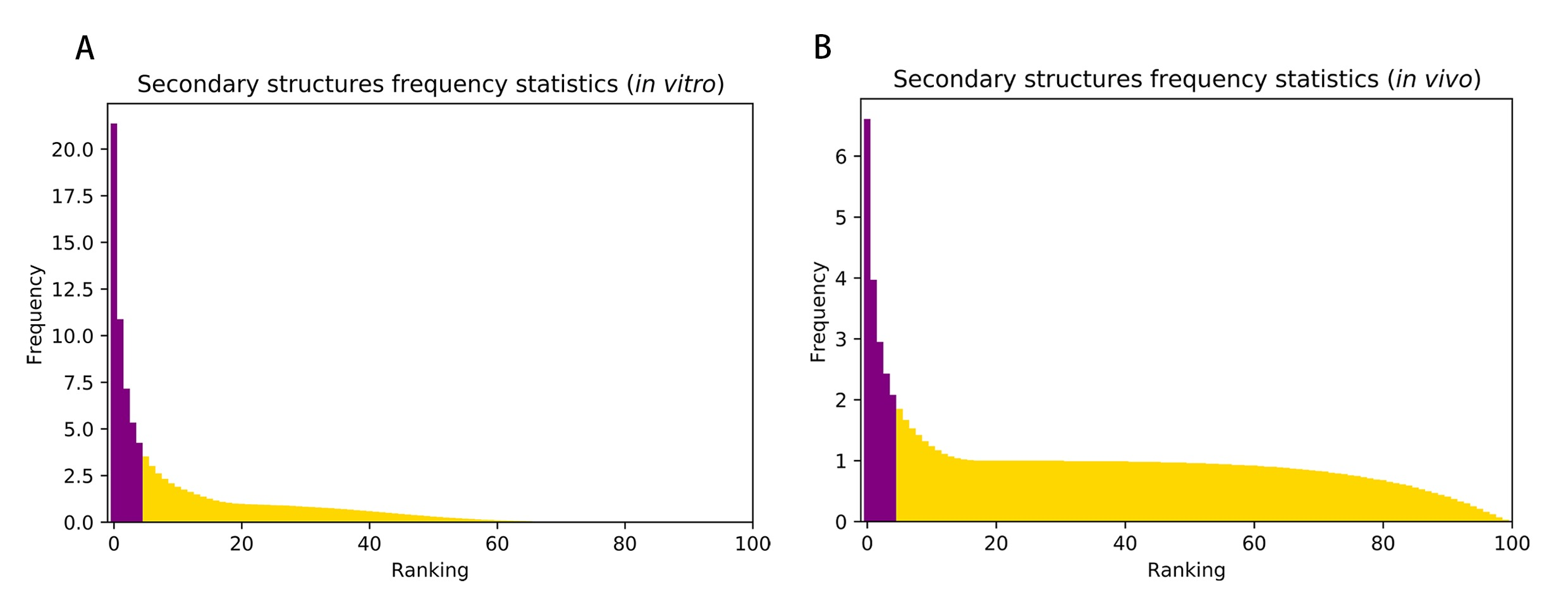

Supplement: S1 Fig — Structures are sorted from the most frequent to the least frequent in the x-axis. The y-axis shows the average frequencies of structures across all RNAs in each dataset. Structures ranked at the top T = 5 are colored in purple and the remaining structures are colored in yellow. (TIF) [file pcbi.1007283.s005.tif]

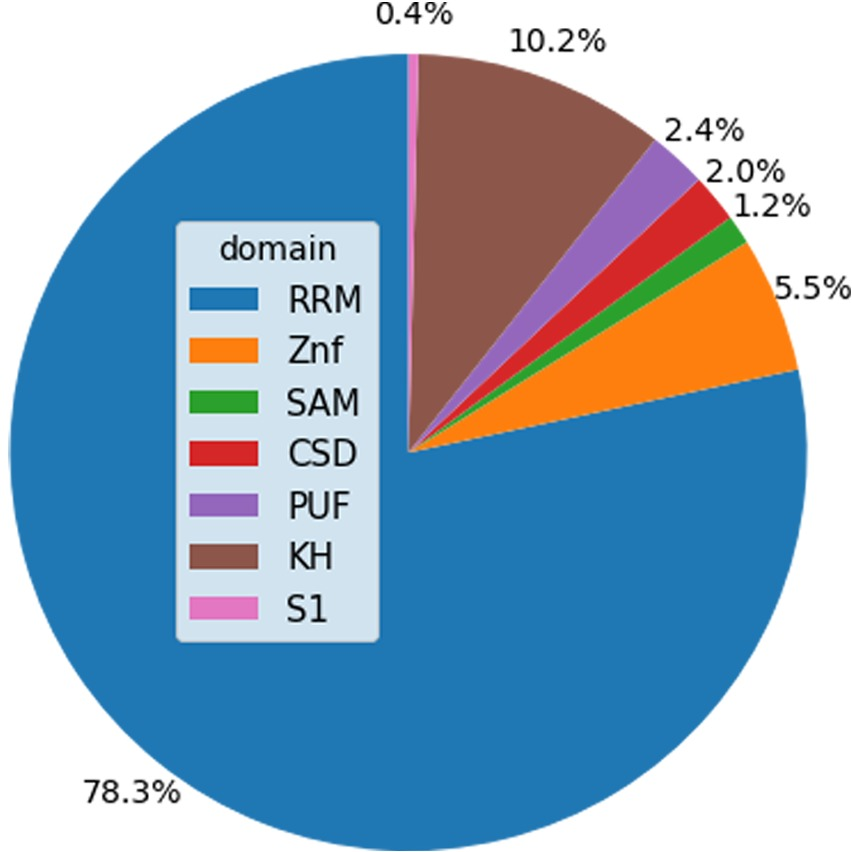

Supplement: S2 Fig — (TIF) [file pcbi.1007283.s006.tif]

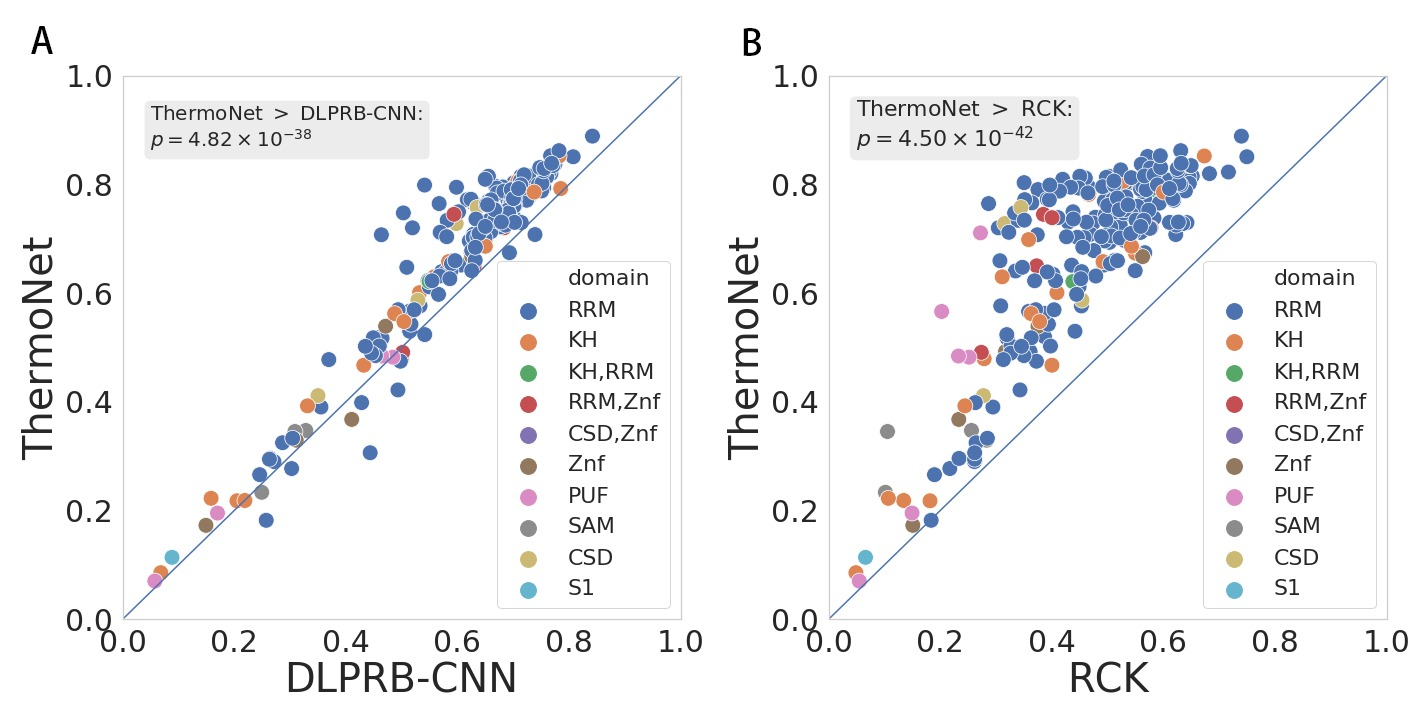

Supplement: S3 Fig — Each point in the scatter plots represents an experiment in the RNAcompete dataset and is labeled with a color specific to its corresponding RNA-binding domain. (TIF) [file pcbi.1007283.s007.tif]

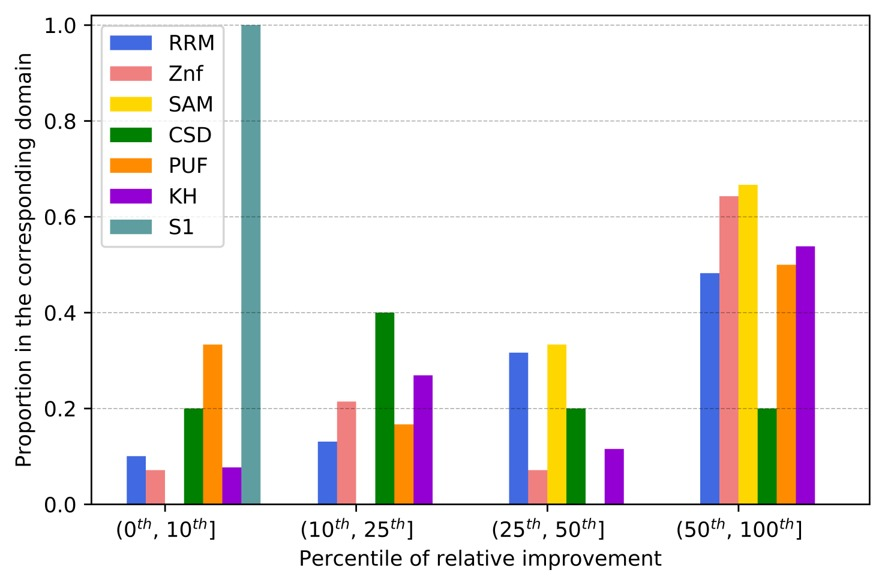

Supplement: S4 Fig — We compute the relative improvement over DLPRB-CNN achieved by our method for each experiment in the RNAcompete dataset. These relative improvements are sorted from the largest to the smallest and then discretized into bins of percentiles (0th, 10th], (10th, 25th], (25th, 50th], (50th, 100th]. The normalized counts of each RNA-binding domain within each bin are shown in the histogram. Note that the S1 binding domain has only one protein hence the relative count of the S1 domain in the (0th, 10th] bin is 1.0. (TIF) [file pcbi.1007283.s008.tif]
